# Effect of Zn and Cu Addition on Mechanical Properties of As-Extruded Mg-3Sn-1Ca Alloy

**DOI:** 10.3390/ma15134438

**Published:** 2022-06-23

**Authors:** Zheng Jia, Bing Yu, Li Fu

**Affiliations:** College of Mechanical Engineering, Shenyang University, Shenyang 110044, China; bingyuxs@sina.com (B.Y.); fuli@syu.edu.cn (L.F.)

**Keywords:** Mg-3Sn-1Ca alloy, extrusion, Mg2Cu phase, texture, mechanical properties

## Abstract

The effects of Zn and Cu addition on the microstructure and mechanical properties of the extruded Mg-3Sn-1Ca alloy were systematically studied. The effects of the grain size, texture, type and distribution of the second phase on the mechanical properties of the alloy were analyzed. The mechanical test results show that the addition of Zn and Cu elements can significantly improve the mechanical properties of the alloy. The as-extruded Mg-3Sn-1Ca-1Zn-1Cu alloy has the best comprehensive mechanical properties, and the UTS, YS and E_L_ are 244 MPa, 159 MPa and 13.4%, respectively. Compared with the Mg-3Sn-1Ca alloy, the UTS and E_L_ of the Mg-3Sn-1Ca-1Zn alloy are increased by 50 MPa and 132%, respectively. However, the UTS of the TXC311 alloy is increased by 55 MPa, but the ductility of the Mg-3Sn-1Ca-1Cu alloy is far less than that of the Mg-3Sn-1Ca-1Zn alloy, which is mainly attributed to the presence of a large amount of hard and brittle Mg2Cu phase in the alloy. Interestingly, the addition of Zn to Mg-3Sn-1Ca-1Cu alloy can improve the elongation of the alloy, which is due to the solid solution strengthening caused by the Zn element and the formation of small MgZnCu phase with Zn element and the consumption of Mg2Cu phase.

## 1. Introduction

Magnesium alloys, as the lightest metal structure material, are widely used in the 3C electronics industry, aerospace, transportation and other fields at present due to their high strength and specific stiffness [1,2]. However, their poor strength and plasticity seriously affect the practical application of magnesium alloys, and it is often necessary to improve the strength and plasticity of alloys by affecting the grain size, texture, second phase category and distribution of alloys through thermal deformation and alloying [3,4]. Among many alloy elements, the low price Sn has attracted much attention in recent years. Studies have shown that Sn has high solid solubility in magnesium and significant precipitation strengthening. Adding 1–2%Sn can form a Mg_2_Sn phase with a high melting point in magnesium and its alloys [5]. Furthermore, the Mg–Sn–Ca alloy has attracted extensive attention in recent years due to its good heat resistance. After adding Ca elements to magnesium alloys, CaMgSn and Mg_2_Sn form heat resistant phases and refine grains, which improves high-temperature resistance [6,7]. Rao et al. [8] studied the hot deformation behavior of Mg-3Sn-1Ca alloy and Mg-3Sn-1Ca alloy under compression and found that CaMgSn particles caused significant back stress during hot deformation. Wang et al. [9] studied the effect of the addition of the trace Ce on the mechanical properties of rolled Mg-1.5Sn-0.5Ca alloy and showed that the addition of minor Ce can refine the eutectic structure between CaMgSn and α-Mg, which can make the eutectic structure more evenly distributed in the Mg matrix and improve the strength of the alloys. Kim et al. [10] studied the effect of extrusion speed on Mg-3Sn-2Ca and Mg-5Sn-2Ca alloys and found that the second phase of both alloys were composed of CaMgSn and Mg_2_Ca phases, and the increase of extrusion speed will coarsen the grains and reduce the mechanical properties.

Zn, an inexpensive element, can significantly refine the grain and improve the corrosion resistance of magnesium alloys [11,12,13]. When studying the microstructure and mechanical properties of as-extruded Mg-8Al-2Sn-xZn (x = 0.5, 1 and 1.5 wt.%) alloy, Wang et al. [14] found that the yield strength and elongation increased with the increase of Zn content. Ha et al. [15] studied the corrosion performance of the extruded Mg-(1–4%) Zn alloy, and the results showed that the addition of 1%Zn had the best protection effect on the matrix, and the Zn dissolved in α-Mg matrix can enhance the protective effect of the passive film on the matrix. Subsequently, Ha et al. [16] also studied the corrosion resistance of Mg-5Sn-(1–4%) Zn alloy, and the results show that Mg-5Sn-1Zn alloy has the best corrosion resistance. The hydrogen evolution rate and corrosion potential increase with the increasing of Zn content. Zn dissolved in α-Mg matrix has a protective effect on the surface film. Many studies have found that adding the Cu element to Mg–Zn alloys can refine the grain and improve alloy strength [17,18]. Mehrab et al. [19] studied the effect of Cu content on the microstructure and properties of Mg-Zn alloys and showed that the formation of MgZnCu intermetallics for the Mg-2Zn-0.1Cu alloy and also the Mg(Zn,Cu)_2_ compounds for the Mg-2Zn alloys with higher Cu contents. Moreover, the hot extrusion was applied for the grain refinement and changing the distribution of intermetallics. Li et al. [20] studied the aging behavior of the as-cast ZC62 magnesium alloy and the research shows that Cu increases the eutectic temperature of the alloy, and the alloy can be treated by solid solution at a higher temperature, and more solute atoms can be dissolved into the matrix, which can significantly improve the aging strengthening effect of the alloy. Zhu et al. [21] studied the effect of the addition of Cu on the mechanical properties of ZK60 alloy, and the results show that the elongation of the alloy can reach more than 9.0% after the addition of Cu because the alloy grain was refined and the alloy plasticity was improved. When the content of eutectic structure in the alloy was too much, it distributed along the grain boundary and divided the matrix. When the stress concentration is high, the mechanical properties of the alloy will be adversely affected. Tang et al. [22] studied the microstructure, mechanical properties and compressive creep behavior of the as-cast Mg-5Sn-(0–2%) Cu alloy, and the results showed that the mechanical properties of the Mg-5Sn-1.0Cu alloy reached the highest.

Therefore, the solid solution of Zn element can improve the strength and plasticity of the alloy, while the Cu element can refine the grain, change the type of second phase in the alloy, improve the eutectic temperature of the alloy and increase the aging strengthening ability. However, the effect of Zn and Cu addition on the properties of Mg-3Sn-1Ca alloys is rarely reported. In order to further improve the application field of Mg-3Sn-1Ca alloys, Mg-3Sn-1Ca, Mg-3Sn-1Ca-1Zn, Mg-3Sn-1Ca-1Cu and Mg-3Sn-1Ca-1Cu-1Zn alloys were designed in this paper. The changes of microstructure and mechanical properties of the four alloys were systematically analyzed.

## 2. Experimental Procedure

In this experiment, Mg-3Sn-1Ca, Mg-3Sn-1Ca-1Zn, Mg-3Sn-1Ca-1Cu, Mg-3Sn-1Ca-1Cu-1Zn were prepared by use industrial pure Mg (99.9 wt.%), pure Sn (99.9 wt.%), Mg-25%Ca master alloy, pure Zn (99.9 wt.%), pure Cu (99.9 wt.%), four alloy samples are denoted below as TX31, TXZ311, TXC311, TXZC3111, respectively. Firstly, put pure magnesium into the preheated crucible, and then heat the crucible to 750 °C. After the magnesium is completely melted, remove the slag, add Cu, reduce the temperature to 710 °C. After holding for 10 min, continue to remove slag, add Mg-25%Ca, Sn and Zn, stir for 2~3 min, and then pour into the φ65 × 240 mold under the protection of mixed gas (CO_2_: SF_6_ = 99:1) after holding for 20 min. After cooling to room temperature, the ingot is homogenized (400 °C × 24 h). The homogenized ingot is processed into a billet with a φ47 × 100 mm by lathe, and then backward extruded into a bar with a diameter of 12 mm on a 300 ton vertical hydraulic press. The extrusion temperature was 300 °C, the extrusion speed was 1 mm/s.

**Figure 1:**
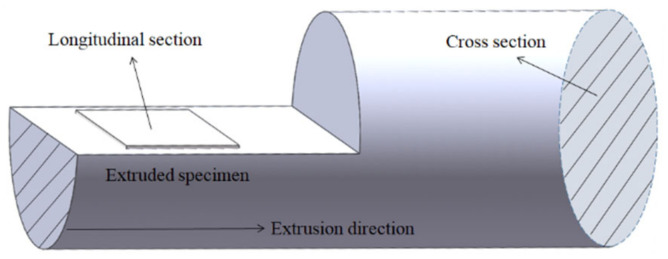
Metallographic sampling location.

The chemical composition of as-cast alloy was analyzed by inductively coupled plasma atomic emission spectrometer (ICP-AES), and the results are shown in Table 1. The sampling location of the alloy microstructure is shown in Figure 1. Shimadzu 700 X-ray diffraction analyzer (XRD) was used to analyze the phase (the target is Cu; the experimental voltage and current were 40 kV and 30 mA respectively; the experimental scanning angle was 20–90°; and the scanning speed was 4°/min). The metallographic samples were sanded to 5000#, and then mechanically polished with 0.5 μm diamond grinding paste. The polished samples were etched with 3 g picric acid, 3 mL glacial acetic acid, 50 mL ethanol and 5 mL deionized water for 5~10 s. The morphology of the second phase particles was observed by S4800 scanning electron microscope (SEM), and the element composition was detected by energy dispersive spectrometer (EDS). The micro crystallographic orientation information of the samples was obtained by electron backscatter diffraction technique in S4800 scanning electron microscope, and the data were analyzed by Channel 5.

## 3. Results

### Microstructure Characterization

**Figure 2:**
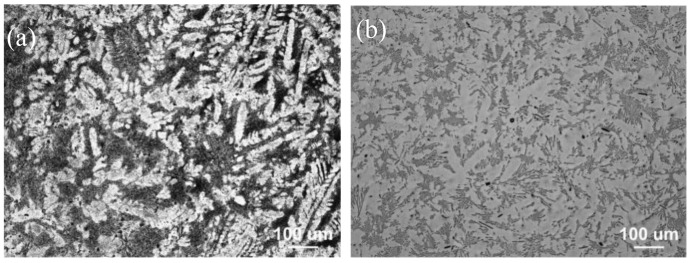

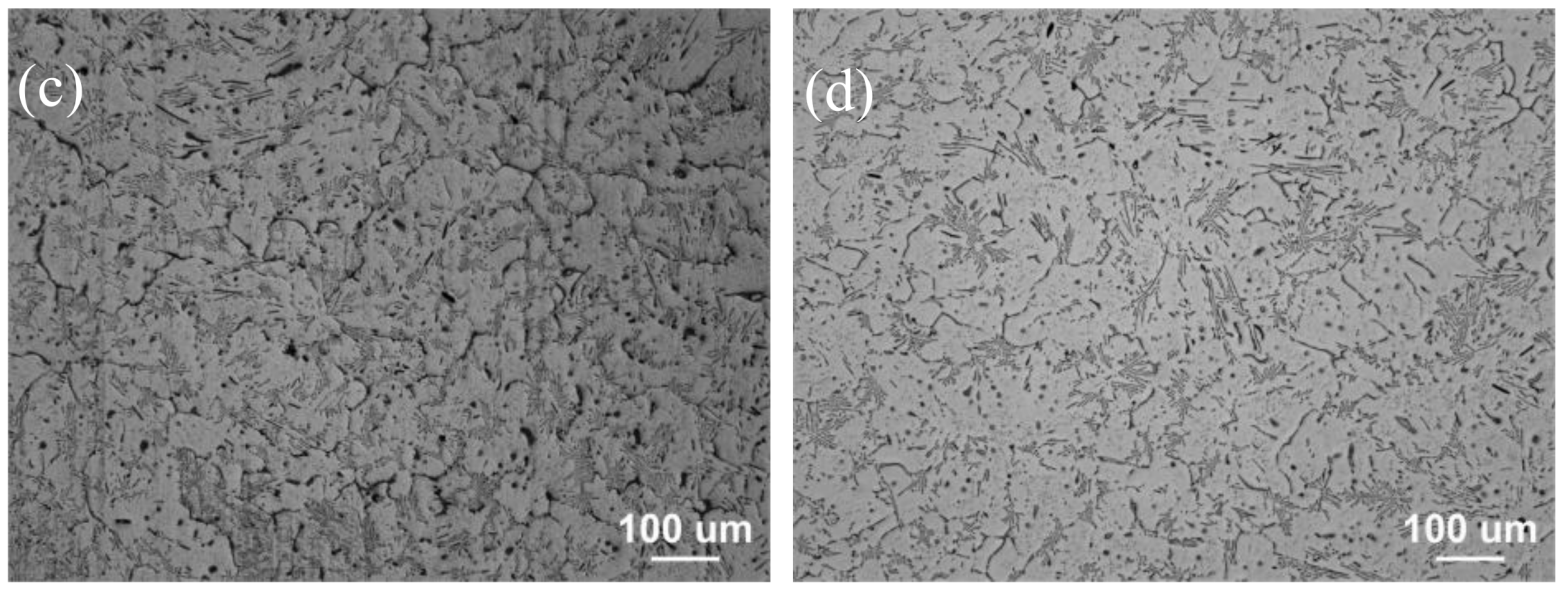
OM images of as-cast alloys: (**a**) TX31; (**b**) TXZ311; (**c**) TXC311; (**d**) TXCZ3111.

Figure 2 shows the optical microstructure (OM) images of the as-cast alloys. It is evident that TX31 ternary alloy has coarse dendrites structure, and the average grain size exceeds 1000 μm (see Figure 2a). After adding Zn and Cu alone, the coarse dendrites were broken and the grain were significantly refined to 500 ± 27 μm (see Figure 2b,c). With the addition of Zn and Cu, the average grain size is further refined to 391 ± 30 μm and the distribution of the second phase is more uniform (see Figure 2d).

**Figure 3:**
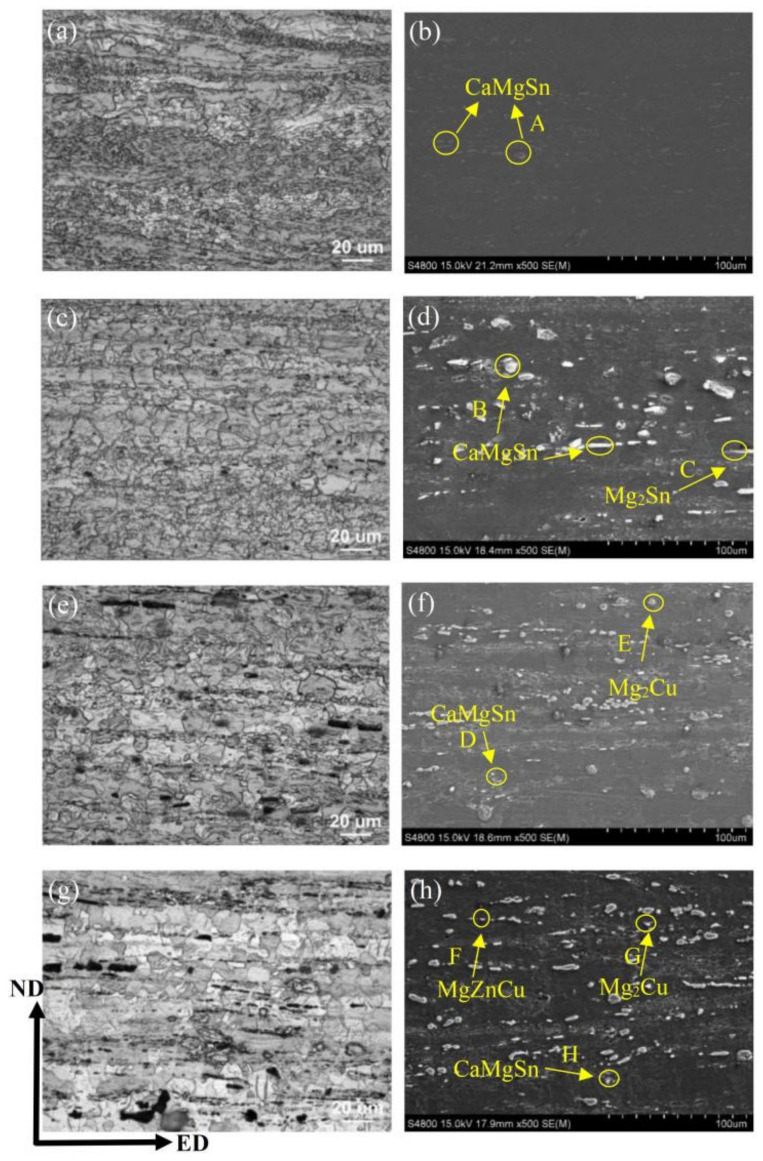
Microstructure and SEM of as-extruded alloys, (**a**,**b**) TX31; (**c**,**d**) TXZ311; (**e**,**f**)TXC311; (**g**,**h**) TXCZ3111.

**Figure 4:**
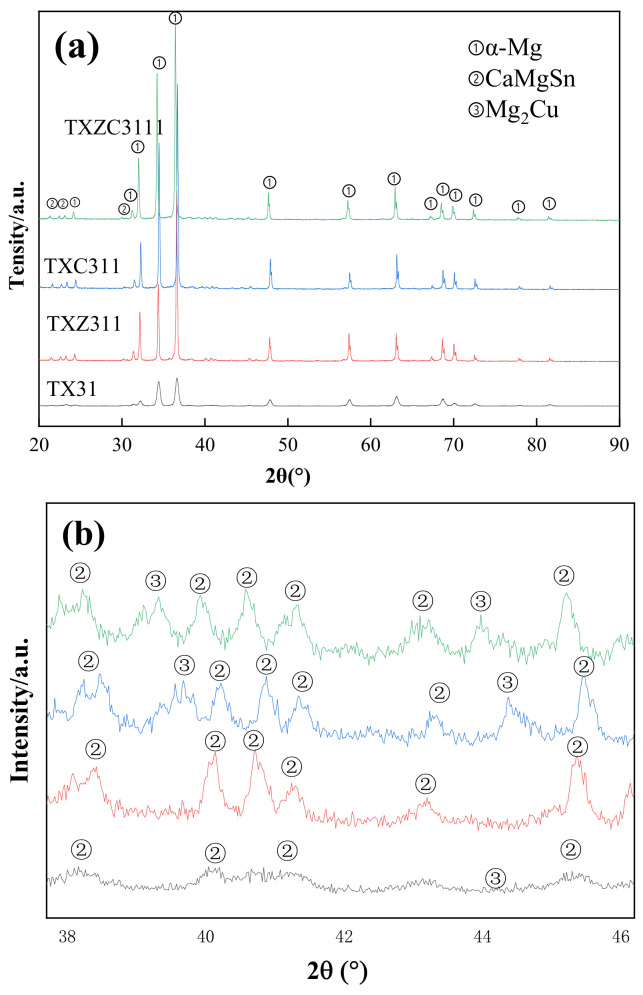
XRD patterns of the as-extruded alloys, (**a**) scan angle 20°~90°; (**b**) scan angle 37°~47°.

Figure 3 shows the OM and SEM images of the as-extruded alloys. Image-Pro Plus software was used to count the area fraction and size of the second phase, as shown in Table 2. EDS results are shown in Table 3. In the as-extruded alloys, dynamic recrystallization occurs obviously, and the broken second phase is linearly distributed along the ED direction. The XRD results are shown in Figure 4. It is found that CaMgSn phase exists in four alloys, and Mg_2_Cu phase is formed after adding Cu. Combined with EDS results, the existence of CaMgSn phase and Mg_2_Cu phase can be proved. In the TX31 alloys, there are large plastic deformation and a small amount of dynamic recrystallization, showing a bimodal texture of fine grain mixed with coarse grain. In contrast, TXZ311, TXC311 and TXZC3111 alloys show more uniform equiaxed grains. Combined with XRD and SEM/EDS, the second phase of the as-extruded TX31 alloy is only composed of granular CaMgSn phase. The area fraction of the second phase in the TXZ311 alloy increased significantly, and there were a large number of coarse CaMgSn phases. The EDS results showed that the atomic ratio of Mg/Sn at point C was close to 1:1, so it was speculated that there was a small amount of Mg_2_Sn phase in the TXZ311 alloy. This shows that the addition of Zn makes the second phase of the alloy coarse without the formation of intermetallic compounds, but the solid is dissolved in α-Mg matrix. Before that, Tang et al. [23] also found the same when studying the Mg-5Sn-xZn alloy, that is, the solid solution of Zn will coarsen the second phase in the magnesium alloy. In addition to massive CaMgSn phase, spherical Mg2Cu phase was found in the TXC311 alloy. There are still a lot of CaMgSn and Mg_2_Cu phases in TXCZ3111 alloy. The results of EDS show that the Zn/Cu ratio at F point is close to 1:1, which is speculated as MgZnCu phase. However, no Mg2Sn phase in the TXZ311 alloy and MgZnCu phase in the TXZC3111 alloy were not detected by XRD due to their amount is so low. The second phase of the four alloys basically exists as CaMgSn phase. Coincidentally, the addition of Ca to Mg-5Sn alloys has previously been reported to contribute to the formation of a more thermally stable CaMgSn phase rather than the Mg2Sn or Mg2Ca phase. The study also shows that the mass ratio of Sn/Ca has a strong effect on the phase composition of Mg–Sn–Ca alloys. When the mass ratio is 3:1, almost all Ca combines with Mg and Sn to form a CaMgSn phase [24].

**Figure 5:**
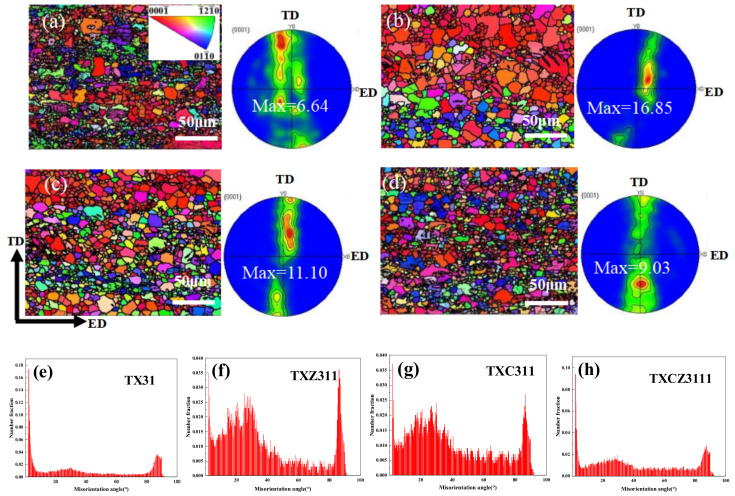
EBSD IPF maps and pole figures of the four extruded alloys, (**a**,**e**) TX31; (**b**,**f**) TXZ311; (**c**,**g**) TXC311; (**d**,**h**) TXCZ3111; (**e**,**f**,**g**,**h**) Misorientation angle distribution of four extruded alloys.

**Figure 6:**
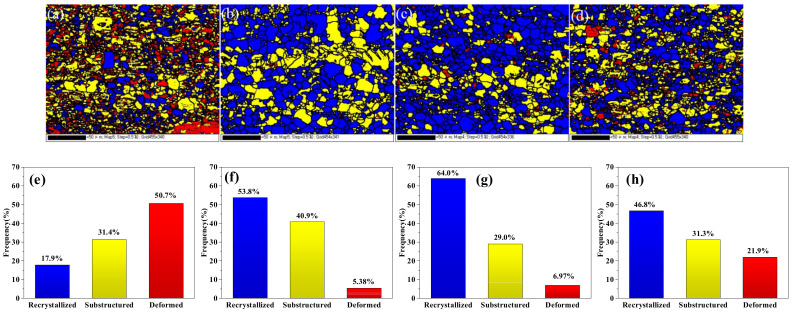
EBSD Map of grain distribution of different types, (**a**) TX31; (**b**) TXZ311; (**c**) TXC311; (**d**) TXCZ3111 (blue-recrystallized, yellow-substructured, red-deformed); (**e**), (**f**), (**g**) and (**h**) frequency of the different types of grains shown in (**a**) (**b**), (**c**) and (**d**), respectively).

Figure 5a–d shows the inverse pore figures (IPFs) maps and pore figures of the as-extruded alloys. Different colors in the IPF figure indicate different grain orientations. The grain size of the as-extruded alloy is obviously more refined than an as-cast alloy. But according to the analysis in Figure 5, the average grain sizes of TX31, TXZ311,TXC311 and TXCZ3111 alloys are 3.5 μm, 5.1 μm, 4.5 μm and 3.5 μm, respectively. For the extruded alloy, the addition of Zn and Cu did not refine the grain. In addition, it is found that the addition of Zn and Cu, respectively, enhances the basal strength of the TX31 alloy, and the basal texture strength of the TXZ311 alloy is the highest, which is 16.85. Compared with TXZ311 and TXC311 alloys, when Zn and Cu are added, the basal texture strength decreases significantly, which is 9.03. Figure 6 presents the EBSD mappings of different grain types in extruded TX31, TXZ311, TXC311 and TXZC3111 alloys, which show recrystallized regions (blue areas), sub-grains (yellow areas), deformed regions (red areas), in their corresponding fraction. The DRXed fraction of the TX31 alloy is only 23.8%. After Zn is added to the TX31 alloy, the DRXed fraction is 56.5%, the deformation regions are significantly reduced. After Cu is added to the TX31 alloy, the DRXed fraction increases to 68%. Compared with TXZ311 and TZC311 alloys, the DRXed fraction of the TXCZ3111 alloy is reduced to 49.6%. Figure 5e–h shows the misorientation angle distributions. The low angular grain boundaries (LAGBs) fractions of the four alloys are 23.5%, 1.4%, 1.4% and 3.6%, respectively. After adding Zn and Cu alone, the fraction of LAGBs decreased and the fraction of high angular grain boundaries (HAGBs) increased. After adding Zn and Cu, the fraction of LAGBs increased and the fraction of high angular grain boundaries (HAGBs) decreased.

**Figure 7:**
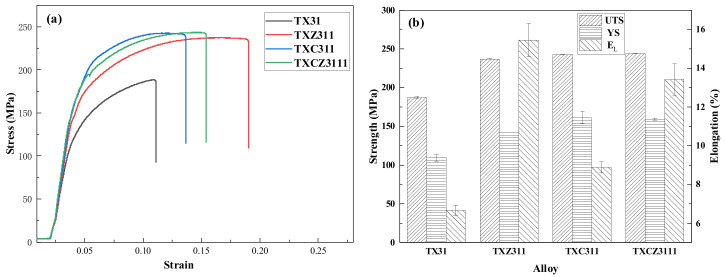
Mechanical properties of four extruded alloys at room temperature, (**a**) stress–strain curve; (**b**) mechanical property diagram.

Figure 7 shows the stress–strain curves and mechanical properties of the four as-extruded alloys at room temperature. The ultimate tensile strength (UTS) of TX31, TXZ311, TXC311 and TXCZ3111 alloys is 187 ± 1.2 MPa, 237 ± 0.1 MPa, 243 ± 0.5 MPa and 244 ± 0.5 MPa, respectively. The yield strength (YS) is 110 ± 4.3 MPa, 142 ± 0.3 MPa, 161 ± 8.4 MPa, 159 ± 1.3 MPa, and the elongation (EL) is 6.7 ± 0.3%, 15.5 ± 0.8%, 8.9 ± 0.3%, 13.0 ± 0.8%, respectively. The mechanical properties of the TX31 alloy were significantly improved by Zn and Cu addition. After adding Zn to the as-extruded TX31 alloy, the alloy strength and elongation increased by 26.7% and 132.0%, respectively. After adding the Cu element, the strength of the TX31 alloy increased by 29.6%, while the elongation increased by only 33.3%. Compared with the as-extruded TX31 alloy, the combined addition of Zn and Cu further improves the strength of the alloy, and the elongation decreases slightly. It shows that Zn can improve the strength and plasticity of the as-extruded TX31 alloy.

## 4. Discussion

### 4.1. Microstructure Analysis

As for the as-cast TX31 alloy, as shown in Figure 2a, its structure is typically dendritic, which is due to the high growth limiting factor and slow diffusion rate of Ca element in the alloy. Therefore, the enrichment of Ca atoms in front of solid/liquid interface leads to dense structural supercooling, which limits the grain growth and leads to the formation of dendrites [25]. The addition of Zn and Cu significantly refines the grain size of the as-cast TX31 alloy, and the increase of grain boundary area provides more nucleation sites for DRX, which is beneficial to the DRX of the alloy during extrusion. As can be seen from the Figure 6 that the addition of Zn and Cu significantly improves the DRXed fraction of the as-extruded TX31 alloy.

In general, DRX involves two processes: nucleation and growth. According to the recrystallization theory of stimulated nucleation of second phase particles, the second phase can promote or inhibit the nucleation of DRX, which mainly depends on the size of second phase particles. Large second phase particles (>1 μm) can promote DRX by increasing dislocation density as an effective nucleation sites, whereas fine second phase particles (<1 μm) can delay DRX by inducing the grain boundary pinning effect [26]. In this study, the second phase of the TX31 alloy is relatively fine, the dynamic recrystallization degree of TX31 alloy is relatively low. With the addition of Zn, the second phase particles in the TX31 alloy are obviously coarsened (up to ~200 μm), and the area fraction of the second phase increases significantly, as shown in Figure 3d. Compared with the TXZ311 and TXC311 alloys, the grain size of the TXCZ3111 alloy is refined, the DRXed fraction is slightly reduced, and the deformation regions is increased. It is considered that the migration of grain boundaries during deformation is hindered by the precipitation of a small amount of fine MgZnCu phase.

### 4.2. Texture Analysis

In addition, the texture of the as-extruded TX31 alloy is weak, only 6.64. The analysis shows that because the deformation texture formed in the extrusion process and the recrystallization texture in the dynamic recrystallization process appear simultaneously or alternately so that the two types of textures cannot be fully developed, resulting in the weak texture formed after thermal deformation (as shown in Figure 5a). After adding Zn and Cu elements, the texture strength of the basal plane increases with the increase of grain size, which is due to the reduction of texture components and more concentrated orientation caused by the annexation of small grains in the process of grain growth (as shown in Figure 5b–d).

The distribution of LAGBs and HAGBs also has an important impact on the strength of the alloy (as shown in Figure 5e–h). It is found that there are a large number of LAGBs (23.5%) in the TX31 alloy after extrusion deformation. After adding Zn and Cu, the number of LAGBS in the alloy increases sharply due to the improvement of recrystallization degree. The LAGBs of TXZ311 and TXC311 alloys decreased to 1.4%, and that of the TXCZ3111 alloy increased slightly to 3.6%. The results show that under the action of applied stress, HAGBs show higher mobility, so grain boundary sliding is easier to start quickly, resulting in good plasticity of the alloy. This is consistent with the plastic changes of the four extruded alloys. However, the plasticity of the TXC311 alloy is much lower than that of the TXZ311 alloy, which can be explained by the Schmid factor of four alloys.

**Figure 8:**
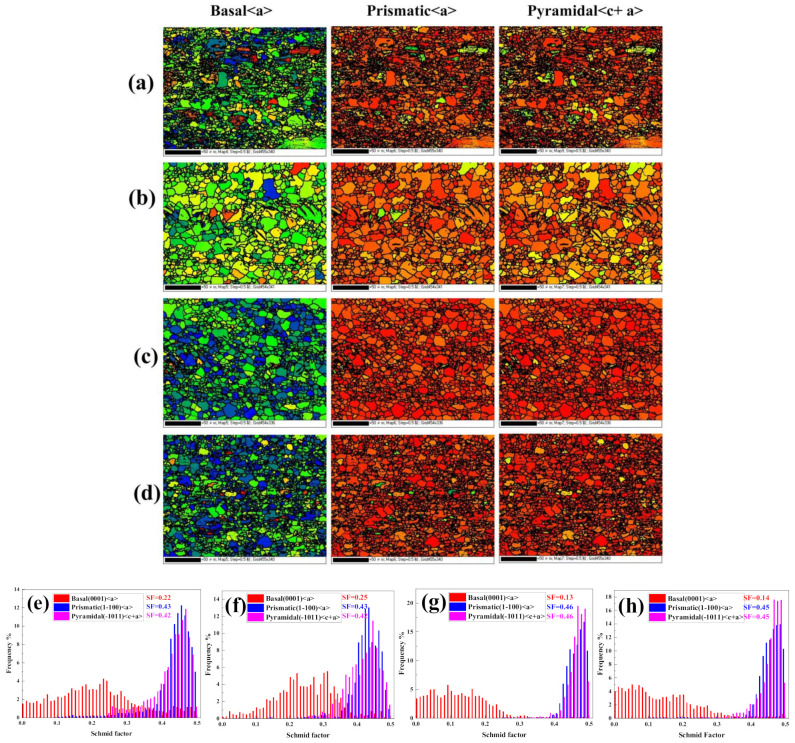
Schmid factors maps and distribution for (0001)/<11-20> basal slip, (10-10)/<11-20> prismatic slip, (10-11)/<11-23> pyramidal slip of as-extrudedTX31 (**a**,**e**); TXZ311 (**b**,**f**); TXC311 (**c**,**g**);TXCZ3111 (**d**,**h**) alloy, respectively.

In order to study the main deformation mechanism of the four extruded alloys in this experiment, Figure 8 shows the Schmid factor maps and distribution of basal <a> slip, prismatic <a> slip and pyramidal <c+a> slip of the four as-extruded alloys, respectively. Generally, SF represents the feasibility and activation degree of a slip mode in the loading direction. The higher the average SF value, the easier the basal slip is activated, so the lower the yield strength [27,28]. It can be found from the figure that the average Schmid factor of the basal <a> slip of the TX31 alloy is relatively lower than that of prismatic <a> slip and pyramidal <c+a> slip, indicating that non-basal slip is the main deformation mode of the TX31 alloy. The average Schmid factor of the non-basal slip of the TXZ311 alloy does not change, but the average Schmid factor of the basal slip increases, indicating that the CRSS required to activate the basal slip of the TXZ311 alloy decreases, which means that the basal slip of the TXZ311 alloy is easier to activate and shows better plasticity. In TXC31 and TXCZ3111 alloys, the average Schmid factor of non-basal slip increases and the average Schmid factor of basal slip decreases, indicating that it is more difficult to open the basal slip of the two alloys, and non-basal slip is the main deformation mode. Compared with the TXC311 alloy, the average Schmid factor of basal slip of the TXCZ3111 alloy increases, and the average Schmid factor of non-basal slip decreases, which shows that the plasticity of the TXCZ3111 alloy is higher than that of the TXC311 alloy.

### 4.3. Mechanical Property Analysis

Generally speaking, non-basal plane slip is the main deformation form of the magnesium alloy at high temperature. The activation of non-basal plane slip in the as-extruded alloy may be related to the following factors: Firstly, the higher DRXed fraction makes deformation more uniform, and the finer grains reduce the critical resolved shear stress (CRSS) difference between the basal plane and non-basal plane. Secondly, in the process of tensile deformation, the coordination stress of grain boundary promotes the cross slip from basal dislocation to non-basal dislocation [29,30,31]. In this study, the addition of Zn and Cu significantly improved the yield strength and ductility of the TX31 alloy, especially the addition of Zn increased the elongation of the TX31 alloy by 132%. This is because after adding Zn, due to the entanglement between solute atoms and dislocations, solution hardening improves the yield strength of the alloy. At the same time, the addition of Zn solute induces the softening of solid solution, reduces the anisotropy of CRSS between the basal slip system and the non-basal slip system and improves the ductility of the alloy by activating the non-basal slip system [32]. In addition, the yield strength of the as-extruded alloy can be attributed to precipitate strengthening and solid solution strengthening. It can be described by the following formula [33,34,35]:

(1) $$∆\sigma_{y}=M\left(∆\tau_{S}+∆\tau_{P}\right)$$

$∆\sigma_{y}$ is the yield strength increment, *M* represents the Taylor factor, and $∆\tau_{S}$ and $∆\tau_{P}$ represents the CRSS change due to solid solution strengthening and precipitate strengthening, respectively. In the as-extruded TXZ311 alloy, Zn is almost dissolved in magnesium matrix, which is a solid solution strengthening effect. The increase of the area fraction of the second phase in the TXZ311 alloy also has a significant effect on the strength of the alloy. In the TXC311 alloy, the solid solubility of Cu is about 0.5, so it has the effect of solid solution strengthening, forms a spherical Mg_2_Cu phase with Mg matrix and has the effect of precipitation strengthening. In the TXCZ3111 alloy, Mg2Cu phase and a small amount of MgZnCu ternary phase are formed by the compound addition of Zn and Cu, most of Zn and a small amount of Cu are still solidly dissolved in α-Mg matrix, indicating that the precipitation strengthening and solid solution strengthening are remarkable. Therefore, compared with the extruded TX31 alloy, the strengthening of the as-extruded TXZ311, TXC311 and TXCZ3111 alloys can be explained by the synergistic effect of precipitation strengthening and solid solution strengthening.

Generally, the Taylor factor M of magnesium alloys with random texture is about 4.5, while the Taylor factor of magnesium alloys with strong texture is reduced to 2.1~2.5, and tends to pyramidal slip [36]. In this study, according to EBSD data, Taylor factor M of TXZ311, TXC311 and TXCZ3111 alloys was calculated to be 2.69, 2.84 and 2.89, respectively, which corresponds to the alloy strength. In addition, the second phase strengthening is one of the important factors affecting the yield strength of magnesium alloys. The influence of the second phase on the strength is quite complex, and its shape, size and distribution all affect the strength of alloy. The finer the spacing and particle size between the second phase particles, the more obvious the strengthening effect. By observing the second phase particles in the as-extruded alloys (See Figure 3), it can be inferred that the sparsely distributed CaMgSn phase particles cannot effectively promote the high strength of the alloy, but the uniformly distributed massive CaMgSn phase, spherical Mg2Cu phase, and a small amount of the Mg2Sn and MgZnCu phase can hinder the movement of dislocation and lead to dislocation accumulation, so as to improve the strength of the alloy. Thus, increasing the alloy strength. Assuming that the second phase follows Orowan [37], the effect of the second phase on yield strength can be expressed by the following formula:

(2) $$YS\propto f^{1/2}D^{-1}InD$$

where D represents the particle diameter of the second phase and f represents the area fraction of the second phase. Obviously, the increase of the area fraction of the second phase can improve the yield strength of the alloy. The area fraction of the second phase in the TXZ311, TXC311 and TXCZ311 alloys is much higher than that in the TX31 alloy, and the distribution is closer, so the alloy strength is improved.

**Figure 9:**
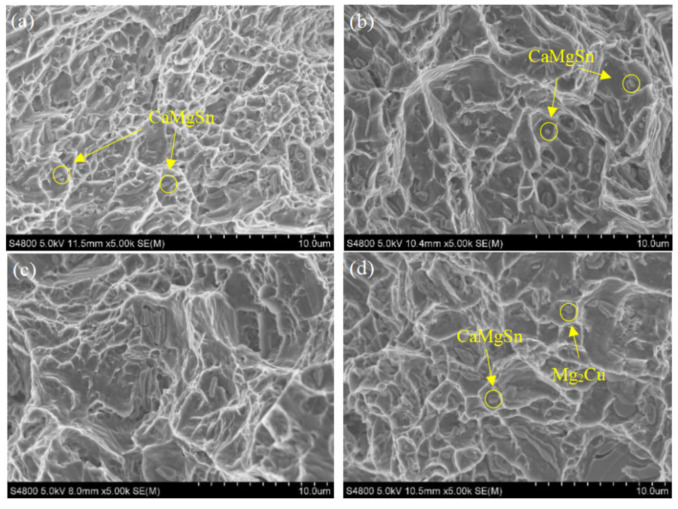
SEM morphology of tensile fracture of as-extruded alloys at room temperature, (**a**) TX31; (**b**) TXZ311; (**c**) TXC311; (**d**) TXCZ3111.

According to the stress–strain curve in Figure 7a, the elongation of the TX31 alloy increased by 132% after adding Zn. According to the observation of microstructure, this is closely related to the number and size of the second phase. In addition, the literature shows that the Zn-modified samples increases the possibility of prismatic slip activation during deformation, which is beneficial to adjust the strain of grain c-axis, and can also inhibit grain boundary cracking to enhance grain boundary cohesion, thus greatly improving the plasticity of the alloy [38,39]. In the Cu-modified samples, Mg_2_Cu phase is formed, and the yield strength is increased by 19.5 MPa, compared with the TXZ311alloy, but the elongation is reduced to 8.89%, which is mainly due to the high hardness and modulus of Mg2Cu phase [40]. The brittle phases are very harmful to the elongation of magnesium alloys because the brittle phase can easily form initial cracks and extend to grains, leading to the reduction of strength. Mg2Cu phase is found at the bottom of the dimple in Figure 9d. Therefore, the Mg2Cu and CaMgSn phase are judged to be detrimental to the plasticity of the alloy. However, when Cu and Zn are added together, some Cu and Zn elements form a fine MgZnCu phase, which leads to the decrease of Zn elements and Mg2Cu phase in the solid solution α-Mg matrix. Therefore, compared with the TXZ311 alloy, the tensile strength and yield strength of the TXCZ3111 alloy increase, while the elongation increases significantly compared with the TXC311 alloy.

In order to verify the relationship between the fracture mechanism and mechanical properties of the alloys, Figure 9 shows the SEM images of the tensile fracture of the four as-extruded alloys. The fracture represents a large number of equiaxed dimples, and the second phase particles are found at the bottom of the dimples. According to the EDS results, it is proved that the particles are CaMgSn phase, indicating that the alloy presents the characteristics of ductile fracture. Equiaxed dimples are microcracks formed at the interface between inclusions, second phase particles and matrix. The adjacent microcracks converge to produce micro-holes, which then becomes cavity growth and increment, and finally connects to form fractures, leaving traces on the fracture surface [41]. This indicates that the CaMgSn phase found in the dimples is the main reason for crack nucleation and propagation. For example, in this study, a large number of small dimples were found in the as-extruded TX31 alloy, with the falling CaMgSn phase inside. This is due to the stress concentration caused by dislocation during deformation, and the incongruous plastic deformation between the second phase and the matrix, resulting in the nucleation, growth and polymerization of dimples during deformation. After adding Zn and Cu, it is found that the dimple size of the fracture is larger and deeper than that of the TX31 alloy, and the falling second phase is significantly reduced. Almost no second phase is observed in the dimple of the TXZ311 alloy, indicating that the strength and toughness of the alloy are improved. From the SEM morphology of the fracture, it can be concluded that the addition of Zn and Cu will lead to significant changes in the tensile fracture of the extruded TX31 alloy, which are consistent with the changes in the toughness of the alloy.

**Figure 10:**
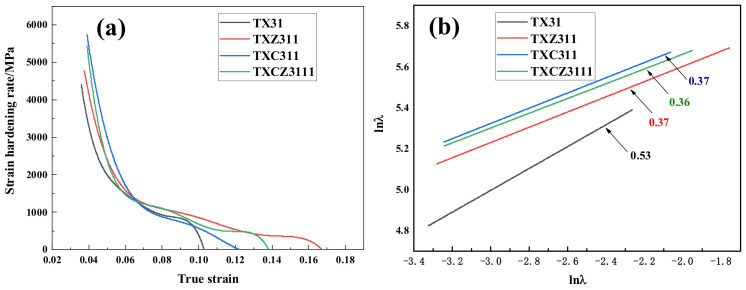
Strain hardening rate (**a**) and strain hardening index (**b**) of four extruded alloys.

In order to analyze the strain hardening behavior of four extruded alloys, the strain hardening rate versus true strain curve and strain hardening index curve were drawn, as shown in Figure 10. The work hardening rate is derived as follows [42]:

(3) $$\theta =(\frac{\delta \sigma }{\delta \varepsilon }{)}_{\dot{\varepsilon }}$$

where δσ, δε and $\dot{\varepsilon }$ are the true stress, true strain and strain rate, respectively. In Figure 10a, the TX31 alloy shows a low strain hardening rate, indicating that the strain hardening behavior of the alloy is poor. Initially, the strain hardening rate of the other three alloys is the TXC311 > TXCZ3111 > TXZ311 alloy. When the strain is 0.06, the hardening ability of the TXC311 alloy decreases significantly. At this phase, TXZ311 and TXCZ3111 alloys show better strain hardening ability. Figure 10b shows the change of the strain hardening index of the four extruded alloys. It shows that the strain hardening index of the TX31 alloy is the highest, but because the yield strength of the alloy is low at this phase, the strain hardening index of the other three alloys has little difference, indicating that there is little difference in the strain hardening ability of the TXZ311, TXC311 and TXCZ3111 alloys. The poor strain rate of the TX31 alloy is due to the existence of a large number of CaMgSn hard brittle phases. In the TXZ311 alloy, the effect of Zn solution strengthening and precipitation strengthening, the anisotropy of basal slip and non-basal slip decreases, and the strength and plasticity increase. After adding Cu alone, spherical Mg_2_Cu phase is formed in the alloy, which is due to the low solid solubility of Cu in magnesium (0.55%). The strength of the TXC311 alloy is further improved under the action of solid solution strengthening and second phase strengthening, but the anisotropy of basal slip and non-basal slip is significantly increased, so the plasticity is relatively lower than that of the TXZ311 alloy. After Zn and Cu are added together, in addition to Mg_2_Cu phase, a small amount of MgZnCu phase is formed in the alloy, which plays the role of second phase strengthening. Compared with the TXC311 alloy, the Schmid factor of basal slip of the TXCZ3111 alloy increases, and its plasticity increases.

## 5. Conclusions

The Mg-3Sn-1Ca alloy is a potential heat-resistant magnesium alloy. In order to improve its strength, this paper systematically studied the effects of Zn and Cu elements on the microstructure and mechanical properties of the extruded Mg-3Sn-1Ca alloy, and the following conclusions can be drawn:

1. In the TX31 alloy, except for a large amount of CaMgSn phase, the addition of Cu forms the spherical Mg_2_Cu phase, the addition of Zn is solidly dissolved in the magnesium matrix, and the combined addition of Zn and Cu can form MgZnCu phase.
2. Due to the existence of a large number of CaMgSn phase and Mg_2_Cu hard brittle phase in the as-extruded TX31 and TXC311 alloys, the plasticity is poor, which can be improved by adding Zn.
3. Among the four as-extruded alloys, the tensile strength and yield strength are TXCZ3111 > TXC311 > TXZ311 > TX31, and the EL is TXZ311 > TXCZ3111 > TXC311 > TX31. The TXCZ3111 alloy has the best comprehensive mechanical properties, with a UTS, YS and EL of 244 MPa, 159 MPa and 13.4%, respectively. The strength of the TXC311 alloy is equivalent to that of TXCZ3111 alloy, but the plasticity is much lower than that of the TXCZ3111 alloy. The TXZ311 alloy has the best plasticity, but its strength is slightly lower than the TXCZ3111 alloy.

## Figures and Tables

**Table 1:** Actual chemical composition of as-cast alloy.

Alloy	Measured Composition(wt.%)				
	Mg	Sn	Ca	Zn	Cu
Mg-3Sn-1Ca(TX31)	Bal.	3.14	1.02	0	0
Mg-3Sn-1Ca-1Zn(TXZ311)	Bal.	3.12	1.02	1.09	0
Mg-3Sn-1Ca-1Cu(TXC311)	Bal.	3.22	1.04	0	1.07
Mg-3Sn-1Ca-1Zn-1Cu(TXCZ3111)	Bal.	3.18	1.04	1.07	1.04

**Table 2:** The area fraction and size distribution of the second phase.

Alloy		Second Phase Size Distribution (μm)			
	Area Fraction of Second Phase(%)	CaMgSn	Mg_2_Sn	Mg_2_Cu	MgZnCu
TX31	7.5 ± 1.0%	5~60 μm	-	-	-
TXZ311	11.0 ± 3.1%	10~200 μm	80~100 μm	-	-
TXC311	8.0 ± 1.4%	58~110 μm	-	30–70 μm	-
TXCZ3111	9.1 ± 1.8%	8~100 μm	-	20~40 μm	20~30 μm

**Table 3:** EDS analysis results.

Point	Chemical Composition(at.%)					Phase Types
	Mg	Sn	Ca	Zn	Cu	
A	57.19	22.98	19.28	-	-	MgSnCa
B	66.50	17.89	14.58	-	-	MgSnCa
C	58.12	41.88	-	-	-	Mg_2_Sn
D	72.90	15.35	11.33	-	-	MgSnCa
E	72.87	-	-	-	27.13	Mg_2_Cu
F	65.36	-	-	16.92	17.72	MgZnCu
G	74.53	-	-	-	25.47	Mg_2_Cu
H	84.38	8.04	7.58	-	-	MgSnCa

## Data Availability

Not applicable.
